# Food-related quality of life in inflammatory bowel disease: measuring the validity and reliability of the Turkish version of FR-QOL-29

**DOI:** 10.1186/s12955-022-02014-9

**Published:** 2022-07-05

**Authors:** Nazlı Nur Aslan Çin, Kevin Whelan, Ayşe Özfer Özçelik

**Affiliations:** 1 Department of Nutrition and Dietetics, Faculty of Health Sciences, Karadeniz Technical University, Trabzon, Turkey; 2 Department of Nutritional Sciences, King’s College London, London, UK; 3 Department of Nutrition and Dietetics, Faculty of Health Sciences, Ankara University, Ankara, Turkey

**Keywords:** Inflammatory bowel disease, Food, Quality of life, Nutrition, Validation

## Abstract

**Purpose:**

Food-related quality of life is considerably impaired in patients with inflammatory bowel disease (IBD) and should be widely measured in research and clinical practice. This study aims to translate the FR-QoL-29 instrument to the Turkish language and evaluate its validity and reliability in Turkish patients with IBD.

**Methods:**

The FR-QoL-29 was forwards and backwards translated into Turkish and the validity and reliability of the FR-QoL-29-Turkish measured at two tertiary hospitals in Ankara, Turkey. Participants completed four questionnaires regarding: sociodemographic; clinical (disease type and activity), and nutritional characteristics (MUST) together with FR-QoL-29-Turkish. In addition, 30 patients repeated the questionnaires after two weeks. collected. Pearson correlation coefficients and Cronbach α were used to assess reliability and validity (p < 0.05).

**Results:**

A total of 180 participants with IBD (78 Crohn’s disease, 102 ulcerative colitis), with a mean age of 45.9 ± 12.5 years, were included. Bartlett's sphericity test was statistically significant (p < 0.001), meeting the prerequisite for factor analysis, and the adequacy of the sample size for factor analysis was confirmed by a high Kaiser–Meyer–Olkin (KMO = 0.92). Validity was confirmed by factor loadings ranging from 0.310 to 0.858. Item-total score correlations ranged from 0.258 to 0.837 and Cronbach’s α coefficient was 0.96 for the whole questionnaire indicating high internal consistency.

**Conclusions:**

FR-QoL-29-Turkish is a valid and reliable measure of food-related quality of life in IBD patients with Turkish language. The FR-QoL-29-Turkish gives a comprehensive overview of the main aspects of food quality of life and can be used as a useful tool in both research and clinical practice.

## Plain English summary

## Introduction

Impaired quality of life and psychological stress are associated with both gastrointestinal and systemic symptoms [1]. Inflammatory bowel disease (IBD) is an inflammatory disorder of the gastrointestinal tract that negatively affects quality of life during periods of both remission and active disease [2]. Despite this, almost half of physicians are not eager to investigate the effects of the disease on the quality of life in IBD [3]. Diet and nutritional information are particularly focused on specific nutritional issues such as enteral nutrition rather than dietary management or psychosocial effects [4, 5]. Food-related quality of life (FR-QoL) refers to the impact of food, nutrition, eating and drinking on important psychosocial aspects of life. For example, the ability to maintain social activities such as eating and drinking, to enjoy food, and to achieve adequate nutrition, and how adapting to these in IBD can impact on the psychosocial aspects of food such as eating with family and eating out [6–8].

Although evidence supporting the role of food and drink in the etiology and exacerbation of IBD is controversial, the symptom-triggering effect of various foods and drinks should not be ignored [9]. Patients can avoid consuming certain foods or food groups according to their impact on symptoms or make important dietary changes such as severely restricting the amounts consumed [2, 10, 11]. The IBD patients may feel excluded from social interactions related to eating and drinking. Moreover, these patients deal with problems such as alienation from friends, family, and colleagues due to the avoidance of social events involving food, which may lead to anxiety and depression [12, 13].

Although several studies have examined the deterioration of health-related quality of life in IBD [14, 15], few nationwide studies were related specifically to FR-QoL, in part due to the previous absence of a tool to measure this important phenomenon. Recently, interviews with adults with IBD [13] were used to develop an instrument to measure FR-QoL in IBD that was then validated in a large psychometric evaluation [16]. The instrument, the FR-QoL-29, is written in English and consists of 29 items that people with IBD rate using a Likert scale. It has been used to show impairments in FR-QoL, and their associated factors, in patients with IBD in a range of English-speaking countries, including the United Kingdom [17, 18], United States [12], Australia [19] and New Zealand [20].

Thus far there have been no studies that have measured the validity and reliability of a translated version of the FR-QoL-29 instrument, and thus its ability to validly and reliably measure FR-QoL in non-English speaking countries is unknown. There is a clear necessity to customize this FR-QoL-29 instrument to the Turkish population considering the cultural and language differences. Therefore, this study aims to translate the FR-QoL-29 to the Turkish language and evaluate its validity and reliability in Turkish patients with IBD.

## Methods

### Turkish adaptation protocol

Permission to translate the FR-QoL-29 was obtained from the copyright holders (King’s College London). The FR-QoL-29 was translated into Turkish using the forward–backward method [21]. Two translators who are fluent in English independently translated the questionnaire into Turkish and both versions were checked, and any inconsistencies were resolved collaboratively by the research team. It was then translated back into English by another bilingual speaker who was unfamiliar with the English version to confirm the accuracy of translation.

A group of seven nutritionists, a psychiatrist, and a gastroenterologist tested the content validity of the translated version of FR-QoL-29. Language experts were asked to rate the simplicity, clarity, relevance, and necessity of each question. The scale was tested with 10 IBD patients for any major changes by the research team, and the questionnaire was finalized after necessary minor corrections were applied to produce the final Turkish version (FR-QoL-29-Turkish).

### Study population

Data were collected by face-to-face interview method from 180 patients with IBD who were examined at the gastroenterology outpatient clinic of two tertiary hospitals in Ankara, Turkey between October 2018 and October 2020. Patients aged 20–65 years, diagnosed with IBD, Turkish native, and currently living in Turkey were recruited for the study. Pregnant and/or lactating women were excluded. The study protocol was approved by the Research Ethics Committee of XXX (protocol number: 56786525-050.04.04/112994) and the Helsinki Declaration principles were followed in the research. Each participant was informed about the contents of the study prior to the survey and signed an informed consent form which indicated voluntary participation in the research.

### Construct validity

The construct validity of the scale was assessed using factor analysis for IBD patients. Factor analysis theory states that the sample size should be at least 5 times the number of items [22], and therefore a minimum of 145 participants was required to obtain an adequate sample size for this 29-item scale. A total of 180 IBD patients' responses to allow for missing data.

Data collection included sociodemographic characteristics (age, gender, smoking status, alcohol consumption), clinical characteristics (disease type, disease activity), nutritional characteristics (risk of malnutrition, anthropometry).

Disease activity was evaluated by the Harvey–Bradshaw index in Crohn’s disease (CD) or Partial Mayo index in ulcerative colitis (UC). Active disease in CD was defined as a Harvey–Bradshaw index score > 4 points [23], while active disease in UC was defined as a partial Mayo score > 2 points [24]. Risk of malnutrition was calculated using the Malnutrition Universal Screening Tool (MUST) assessed by a specialist dietitian. The MUST is a 3-step screening tool validated to assess the nutritional status of populations and classifies patients as low nutrition risk (score 0), medium nutrition risk (score 1) and, high nutrition risk (> 1) [25].

Patients were asked to complete the translated version of the FR-QoL-29. The original questionnaire contains 29 questions on a 5-point Likert scale of “1” (definitely agree) to “5” (definitely disagree) and consists of one dimension. Since four items had positive expressions, reverse coding is performed for these questions (8, 9, 24, 25 questions). Each item score is summed and therefore the lowest obtainable score is 29 (indicating poor FR-QoL) and the highest obtainable score is 145 (indicating good FR-QoL) [16]. The exploratory factor analysis (EFA) was performed using varimax rotation principal component analysis to test the factor structures of 29 questions, following the methods used in the original validation study of the original English validation study [16]. The Kaiser–Meyer–Olkin (KMO) test and Bartlett's sphericity test were performed to test sample adequacy.

### Internal consistency and test–retest reliability

Data from the 180 patients were used to perform item analysis and reliability coefficient (Cronbach's α) were used to measure internal consistency. Test–retest reliability was measured in a subgroup of 30 patients who completed the FR-QoL-29-Turkish two weeks after the first completion.

### Data analysis

Confirmatory Factor Analysis (CFA) was performed using the R-Project program [26] and Lavaan package [27]. All other the statistical analyses were performed using SPSS version 25.0 (IBM Corp, Armonk, NY, USA). Model fit was evaluated using the chi-square, root mean error of approximation (RMSEA), goodness fit index (GFI), and comparative fit index (CFI). Chi-square p-value > 0.05, RMSEA < 0.08, GFI and CFI > 0.9 were considered acceptable values. The factorial structure of the FR-QoL-29-Turkish was examined by EFA. The standardized parameter Cronbach’s α was used for the internal consistency. The statistical significance level was set at p < 0.05.

## Results

One-hundred eighty participants between the ages of 20–65 diagnosed with IBD were included in the study (Table 1). The mean (SD) age of the patients was 45.9 (12.5) years. More than half were male (108, 60.0%) and had UC (102, 56.7%). Overall, 37 (20.6%) stated that they applied some form of diet therapy previously and 84 (46.7%) stated that they applied previous history of surgery related to IBD (Table 1).

**Table 1:** Demographic and clinical characteristics of the 180 patients with IBD

Demographic	Mean ± SD or n (%)
Age (years)	45.9 ± 12.5
Gender	
Female	72 (40.0%)
Male	108 (60.0%)
BMI	25.1 ± 4.1
Underweight	3 (1.7%)
Normal	89 (49.4%)
Overweight	63.0 (35.0%)
Obese	25 (13.9%)
*Disease type*	
Crohn’s disease	78 (43.3%)
Ulcerative colitis	102 (56.7%)
*Clinical course*	
Age at diagnosis, years	36.5 ± 12.5
Years since diagnosis	9.4 ± 7.4
Previous history of surgery	84 (46.7%)
Previous history of dietary treatment for IBD	37 (20.6%)
*Disease activity*	
Self-reported active disease	41 (22.8%)
Crohn’s disease activity (HBI) (n = 78)	3.4 ± 2.9
Crohn’s disease number active (HBI > 4) (n = 78)	18 (23.1%)
Ulcerative colitis activity (Partial Mayo) (n = 102)	1.7 ± 2.6
Ulcerative colitis number active (Partial Mayo > 2) (n = 102)	23 (22.5%)

### Construct validity

The EFA was carried out by principal components analysis (Table 2). The suitability of the analysis was confirmed by identified indicators of the high degree of interrelationship between the variables: Bartlett’s test of sphericity was χ2 = 1308; p < 0.001 and the Kaiser–Meyer–Olkin index was 0.98. The FR-QoL-29-Turkish revealed unidimensionality similar to the original scale in English. The factor load, which shows the relationship of each item with the total score, was over 0.30, and this one factor accounted for 50.98% of the variance.

**Table 2:** Results of explanatory factor analysis of the FR-QoL-29-Turkish in 180 patients with IBD

Items		Factor 1
In the past 2 weeks		
1	I have regretted eating and drinking things which have made my IBD symptoms worse	.536
2	My enjoyment of a particular food or drink has been affected by the knowledge that it might trigger my IBD symptoms	.608
3	My IBD has meant that I have had to leave the table while I am eating to go to the toilet	.378
4	I have not been able to predict how long it will take for my body to respond to something I have had to eat or drink, due to my IBD	.310
5	Certain foods have triggered symptoms of my IBD	.590
6	My IBD has meant that I have been nervous that if I eat something I will need to go to the toilet straight away	.637
7	I have avoided having food and drink I know does not agree with my IBD	.728
8	I have felt relaxed about what I can eat and drink despite my IBD	.797
9	I have felt in control of what I eat and drink in relation to my IBD	.724
10	I have struggled to eat the way that is best for my IBD because of other commitments during the day	.703
11	I have been frustrated about not knowing how food and drink will react with my IBD	.766
12	I have had to concentrate on what I have been eating and drinking because of my IBD	.807
13	I have been worried that if I eat I will get symptoms of my IBD	.771
14	I have felt the way that I eat and drink for my IBD has affected my day-to-day life	.752
15	The way I have had to eat for my IBD has restricted my lifestyle	.730
16	I have had to concentrate on what food I buy because of my IBD	.707
17	It has been on my mind how my IBD will be affected by what I eat and drink	.735
18	My IBD has prevented me from getting full pleasure from the food and drink I have had	.674
19	I have felt that I need to know what is in the food I am eating, due to my IBD	.794
20	I have felt that I have to be careful about when I have eaten, because of my IBD	.805
21	I have had to be more aware of what I am eating, due to my IBD	.803
22	I’ve missed being able to eat or drink whatever I want, because of my IBD	.854
23	I have felt that I would like to be able to eat and drink like everyone else	.817
24	I have been happy to eat and drink around people I do not know despite my IBD	.858
25	I have felt that I have been eating and drinking normally despite my IBD	.818
26	I have found it hard not knowing if a certain food will trigger IBD symptoms	.691
27	My IBD has meant I have had to make an effort to get all the nutrients my body needs	.564
28	I have felt that I haven’t known how my IBD will react to food or drink	.674
29	My IBD has meant that I have had to work hard to fit my eating habits in around my activities during the day	.794
	Eigen value	14.79
	The percentage of variance explanation	50.98

The results of the item-based CFA for the FR-QoL-29-Turkish are shown in Table 3. According to these CFA results, all items were grouped unidimensionally, and were statistically significant as follows: CFI = 0.921, RMSEA: 0.046, GFI: 0.931, and SRMR: 0.050.

**Table 3:** Confirmatory factor analysis for FR-QoL-29-Turkish in 180 patients with IBD

Items	β	STD (β)	z	p
Q1	2.491	0.285	8.730	< 0.001
Q2	1.773	0.204	8.701	< 0.001
Q3	2.569	0.293	8.774	< 0.001
Q4	1.483	0.169	8.788	< 0.001
Q5	1.282	0.148	8.681	< 0.001
Q6	1.869	0.215	8.675	< 0.001
Q7	1.155	0.136	8.518	< 0.001
Q8	1.017	0.121	8.399	< 0.001
Q9	1.244	0.146	8.536	< 0.001
Q10	1.449	0.168	8.600	< 0.001
Q11	1.274	0.150	8.491	< 0.001
Q12	0.892	0.107	8.316	< 0.001
Q13	1.184	0.139	8.520	< 0.001
Q14	1.346	0.157	8.551	< 0.001
Q15	1.341	0.156	8.576	< 0.001
Q16	1.341	0.144	8.536	< 0.001
Q17	1.225	0.134	8.639	< 0.001
Q18	1.160	0.116	8.358	< 0.001
Q19	0.973	0.111	8.324	< 0.001
Q20	0.923	0.112	8.318	< 0.001
Q21	0.932	0.098	8.011	< 0.001
Q22	0.788	0.117	8.222	< 0.001
Q23	0.963	0.065	8.003	< 0.001
Q24	0.522	0.111	8.226	< 0.001
Q25	0.914	0.114	8.614	< 0.001
Q26	0.981	0.133	8.702	< 0.001
Q27	1.160	0.136	8.634	< 0.001
Q28	1.177	0.136	8.467	< 0.001
Q29	1.112	0.131	8.467	< 0.001

Sum scores for the FR-QoL-29-Turkish were significantly positively correlated with age (r = 0.353) and BMI (r = 0.172), and negatively correlated with disease activity (r = -0.281) (Table 4). Improved food-related QoL (higher sum scores on the FR-QoL-29-Turkish) was associated with lower nutritional risk (lower sum scores on the MUST) (r = -0.210).

**Table 4:** Correlation between the FR-QoL-29-Turkish score and selected demographic, clinical, and nutritional measures in patients with 180 patients with IBD

Correlation factors	Pearson correlation (r)	p
Age	.353	p < 0.01**
Gender [male = 0, female = 1]	− 119	0.111
Body Mass Index (BMI)	.172	0.021*
Disease type [UC = 0, CD = 1]	− 087	0.247
Years since diagnosis	.089	0.233
Surgery [no surgery = 0, surgery = 1]	.188	0.012*
Disease activity [remission = 0, active = 1]	− 281	< 0.01**
Harvey–Bradshaw index	− 408	< 0.01**
Partial Mayo Score	− 340	< 0.01**
MUST score	− 210	0.005*

^*^p < 0.05, **p < 0.01. *UC* ulcerative colitis, *CD* Crohn’s disease

### Internal consistency and test–retest reliability

The level of internal consistency, item-total correlations, and Cronbach α internal consistency coefficients among the items of the FR-QoL-29-Turkish are shown in Table 5. The Cronbach α coefficient was 0.96, with a coefficient close to 1 meaning the scale is completely reliable. As explained in Table 4, the corrected item-total score correlation (correlation of all items with the total score) is positive and above 0.40 except for two items (questions 3 and 4). Additionally, no item was removed from the FR-QoL-29 scale since there was no increase in the reliability coefficient when an item was removed (Table 5).

**Table 5:** Results of the internal consistency analysis of the FR-QoL-29-Turkish in 180 patients with IBD and test–retest reliability in 30 patients with IBD

Items	Corrected-item-total score correlation	Cronbach’s Alpha if item deleted	Test–retest reliability, Correlation’s coefficients r
Q1	.496	.964	.712*
Q2	.568	.963	.833*
Q3	.333	.965	.697*
Q4	.258	.965	.438*
Q5	.555	.963	.815*
Q6	.602	.963	.848*
Q7	.702	.962	.879*
Q8	.770	.962	.824*
Q9	.699	.962	.736*
Q10	.666	.962	.686*
Q11	.741	.962	.780*
Q12	.789	.962	.872*
Q13	.748	.962	.802*
Q14	.726	.962	.689*
Q15	.703	.962	.840*
Q16	.679	.962	.732*
Q17	.712	.962	.602*
Q18	.650	.963	.638*
Q19	.773	.962	.740*
Q20	.785	.962	.815*
Q21	.783	.962	.876*
Q22	.835	.961	.912*
Q23	.796	.961	.842*
Q24	.837	.961	.902*
Q25	.793	.961	.832*
Q26	.667	.961	.740*
Q27	.534	.963	.680*
Q28	.646	.963	.736*
Q29	.773	.962	.833*

^*^p < 0.001 (2-tailed)

Pearson correlation coefficients were calculated over the scores of 30 participants who completed the pre-and post-tests at a two-week interval. The correlation of the total score between the two time points was 0.970 (p < 0.001). Correlation coefficients for individual items at the two time points were positive, always above 0.40 and all statistically significant (P < 0.001) (Table 5).

## Discussion

The FR-QoL-29 is a valid and reliable scale developed to evaluate the psychosocial effect of food, nutrition, eating and drinking in individuals with IBD [16]. However, currently, this comprehensive, valid, and reliable measurement tool is only tested in the English language. Hence, we performed a robust translation to produce the FR-QoL-29-Turkish and performed the first ever validity and reliability analysis of a translated version of widely used questionnaire for use in both research and clinical practice.

The adequacy of the sample for factor analysis was determined by the Kaiser–Meyer–Olkin value and data suitability with Bartlett’s sphericity test. In the present study, the Bartlett sphericity test value was found to be statistically significant (p < 0.05), and the Kaiser-Meyer Olkin value was > 0.60 (KMO = 0.92), both fulfilling criteria to perform factor analysis [28].

As a result of factor analysis, FR-QoL-29-Turkish resulted in a 29-item unidimensional scale that explained high levels of the variance in the instrument (50.4%). In this study, all items except two (Q3, Q4) had a factor load of over 0.40. However, when the factor load was above 0.30, this resulted in a unidimensional scale. All items in the original scale had a factor load > 0.40 [16]. It is possible that Turkish IBD patients may have had difficulties in answering Q3 and Q4 due to the cultural disparity between Turkish and western populations.

Previous studies reported that the nutritional and psychosocial aspects of eating and drinking are important for IBD patients [7, 29, 30]. Nutritional status, which is screened using MUST, is lower in many long-term conditions, including IBD, than in the healthy population [25, 31]. The MUST scores of the participants decreased significantly (lower risk of malnutrition) as their FR-QoL-29-Turkish scores increased (improved FR-QoL). This scale, which includes socialization and cognitive and emotional factors related to eating and drinking of individuals with IBD, may assist to complement the general nutritional risk status. Further studies are necessary to evaluate the relationship between FR-QoL-29-Turkish, MUST, and the self-elimination of foods.

A high score on the FR-QoL-29-Turkish scale indicates a high food-related quality of life. Hughes et al., (2016) found that patients in the active phase of IBD had significantly lower FR-QoL-29 scale scores than those in remission, however, there were no significant differences in FR-QoL between UC or CD [16]. In agreement with this, in the current study a significant correlation was found between the FR-QoL-29-Turkish score and disease activity rather than the disease type. Thus, symptom severity appears to play an important role in FR-QoL-29. Symptom severity is important for both quality of life and dietary intervention of IBD patients [12, 13, 17]. Therefore, the application of the FR-QoL-29-Turkish scale to participants both in active and remission phases of IBD may reveal accurate results in future studies.Query

The reliability of an instrument is a prerequisite for it to be valid. The Cronbach's α reliability coefficient, an indicator of the consistency of the items in the scale with the whole scale [32] was high (0.96), with a value of > 0.70 defined as reliable [33]. In the original English validation study, the Cronbach α values of the questionnaire was reported as 0.959[16], therefore the FR-QoL-29-Turkish instrument revealed similar internal consistency characteristics to the original instrument. Thus, the reliability coefficient showing internal consistency shows that the FR-QoL-29-Turkish is highly reliable.

Another method used to determine internal consistency is item analysis [34]. The correlation coefficients between the sub-dimensions and the total score of the FR-QoL-29-Turkish scale ranged from 0.25 to 0.83. The items with weak correlations were excluded through the recommendations in the previous literature, for their removal did not influence Cronbach's α. Another reliability criterion is the test–retest method [35]. Both the pre-and post-correlation coefficients of the FR-QoL-29-Turkish scale items were between 0.43 and 0.91 supporting the test–retest reliability of the instrument over time, whilst the correlation between total sum scores at a two-week interval was very high (0.97).

The present study provides a translated instrument in the Turkish language to evaluate FR-QoL in IBD patients. Moreover, this instrument can guide future research on Turkish IBD patients. Although this paper provided important data on FR-QoL, it also had some limitations. Firstly, we recruited only IBD patients for validation analysis unlike the original study that also included a chronic disease control (asthma) and healthy controls to compare instrument performance [16]. Another study used the scale in both IBD and IBS patients [12]. Further studies with larger sample groups as well as its utility in other disease subgroups should be investigated. Secondly, this study was conducted with IBD patients who applied to two centers in Ankara, which may pose potential bias. Its suitability for use in clinical and research settings (via clinical practice and/or other modes of practice such as face-to-face oral interviews) and a wider variety of populations, including clinical populations, should be evaluated in future studies.

## Conclusion

The results of our analysis assessing the relationship between FR-QoL and IBD in patients in Turkey demonstrated that the FR-QoL-29-Turkish is a valid and reliable tool for use in this disease. The FR-QoL-29-Turkish may be useful in counselling services for patients with IBD who suffer both psychosocial and nutritional problems. The researchers recommend further studies to determine the generalizability of the scale beyond the IBD population. Moreover, future research using this scale is recommended to examine different patient groups with diet patterns or elimination diets.
